# Species-specific antifungal activity of blue light

**DOI:** 10.1038/s41598-017-05000-0

**Published:** 2017-07-04

**Authors:** Wioleta J. Trzaska, Helen E. Wrigley, Joanne E. Thwaite, Robin C. May

**Affiliations:** 1 Institute of Microbiology and Infection and School of Biosciences, University of Birmingham, Birmingham, United Kingdom; 2 NIHR Surgical Reconstruction and Microbiology Research Centre, University Hospitals of Birmingham NHS Foundation Trust, Queen Elizabeth Hospital, Birmingham, United Kingdom; 3 Chemical, Biological and Radiological Division, DSTL, Porton Down, Salisbury, Wiltshire UK

## Abstract

Fungal pathogens represent a significant threat to immunocompromised patients or individuals with traumatic injury. Strategies to efficiently remove fungal spores from hospital surfaces and, ideally, patient skin thus offer the prospect of dramatically reducing infections in at-risk patients. Photodynamic inactivation of microbial cells using light holds considerable potential as a non-invasive, minimally destructive disinfection strategy. Recent data indicate that high-intensity blue light effectively removes bacteria from surfaces, but its efficacy against fungi has not been fully tested. Here we test a wide range of fungi that are pathogenic to humans and demonstrate that blue light is effective against some, but not all, fungal species. We additionally note that secondary heating effects are a previously unrecognized confounding factor in establishing the antimicrobial activity of blue light. Thus blue light holds promise for the sterilization of clinical surfaces, but requires further optimization prior to widespread use.

## Introduction

Invasive fungal infection is a common secondary complication of traumatic injury and can involve a wide-range of fungal species from diverse genera such as *Candida*, *Fusarium*, *Rhizopus* and *Scedosporium*, amongst others^1–3^. Fungal spores present in the environment are easily introduced into wounds after traumatic injury such as motor vehicle accidents, environmental disasters or injuries resulting from military operations^1^. Once established in the host, fungal infections are difficult to treat and are associated with high levels of morbidity and mortality. Thus strategies to decolonize hospital surfaces and surface-exposed wound tissue hold considerable promise for reducing secondary fungal infection.

Exposing microbes to a range of different light wavelengths, in combination with photosensitizing dyes, can effectively inactivate various bacteria, mycoplasma and viruses^4^. Such a combination is called photodynamic therapy (PTD) and has been clinically approved. However, a major challenge for PTD is the need to introduce exogenous photosensitizers into the pathogen^5^. More recently, however, there has been considerable interest in exploiting blue light, which appears to be effective against pathogens without the need for exogenous photosensitizers. In particular, Zhang and colleagues demonstrated the efficacy of blue light against several pathogens, including the intrinsically antimicrobial-resistant species *Acinetobacter baumannii*, in a mouse burn model of infection^5, 6^. Importantly, they also demonstrated that bacteria are more susceptible to blue light than keratinocytes, offering advantages over other currently used topical treatments that are often toxic and ineffective. The proposed mechanism behind the action of blue light is the photoexcitation of endogenous porphyrins, resulting in the production of ROS (Reactive Oxygen Species) and cell death^7^, although this has yet to be formally demonstrated for most blue-light susceptible organisms.

To date, very few fungal species have been tested for sensitivity to blue light. Here we test a range of trauma associated fungal pathogens^1^ and show that many, but not all, are effectively inactivated by this treatment. However, we also demonstrate that some of the antimicrobial activity previously ascribed to blue light may in fact result from secondary heating effects and thus recommend a more detailed characterization of this antimicrobial strategy prior to widespread adoption.

## Results

### Blue light shows antifungal activity against some, but not all, fungal species tested

**Figure 1:**
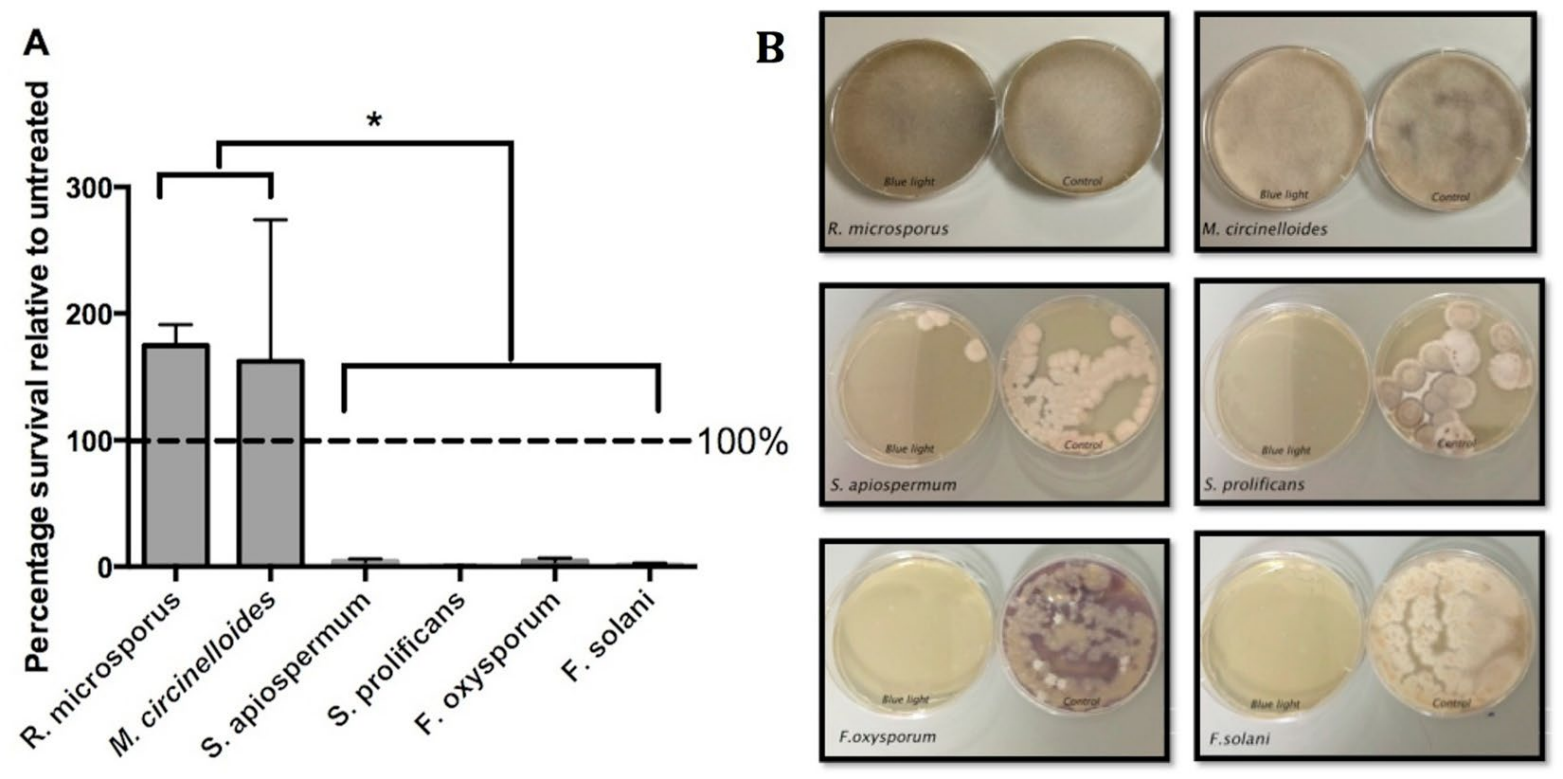
60 min (216J/cm^2^) blue light treatment of fungal spores. Spores were inoculated in PBS treated with blue light and then plated onto appropriate agar plates for enumeration (**A**). Alternatively, 1000 spores were plated onto agar and then exposed to blue light for 60 minutes before being incubated for growth (**B**) Error bars represent standard deviation (n = 3, with three experimental replicates) One-way ANOVA followed by Tukey’s multiple comparisons test shows significant difference (p < 0.05 for all comparisons) in blue light treatment survival between *R*. *microsporus* and *M*. *circinelloides* and other species tested. (**B**) represent images of fungal growth on agar plates following blue light treatment.

For most fungal infections the initiating inocula are fungal spores and disease progression depends on germination and subsequent hyphal invasion of tissue. We therefore exposed spores of six common trauma-associated fungal pathogens (*Rhizopus microsporus*, *Mucor circinelloides*, *Scedosporium apiospermum*, *Scedosporium prolificans*, *Fusarium oxysporum*, *Fusarium solani*) to blue light treatment and then transferred onto agar plates for CFU counting over the following days. Blue light was highly effective against *Scedosporium* and *Fusarium* species, but showed no inhibitory effect but rather enhanced survival relative to controls on the two species of Mucorales tested (Fig. 1A).

To test the potential for blue light to decontaminate solid surfaces, we inoculated fungal spores onto agar and then treated with blue light for 1 hour at room temperature before being allowed to grow for 10 days to determine fungal survival and colony morphology. As with treatment in liquid, blue light exposure on solid (agar) media was highly effective against *Scedosporium and Fusarium* species, but showed no inhibitory effect on the two species of Mucorales tested (Fig. 1B). To ensure that this effect was not related to blue-light induced alteration of the agar surface structure, we performed an additional control by treating agar plates with blue light for one hour and then inoculating with fungal spores, which resulted in normal growth of all fungi tested (Supplementary Figure 2).

### Germination is permanently blocked in most fungal species, but only delayed in Mucorales and *Candida*

**Figure 2:**
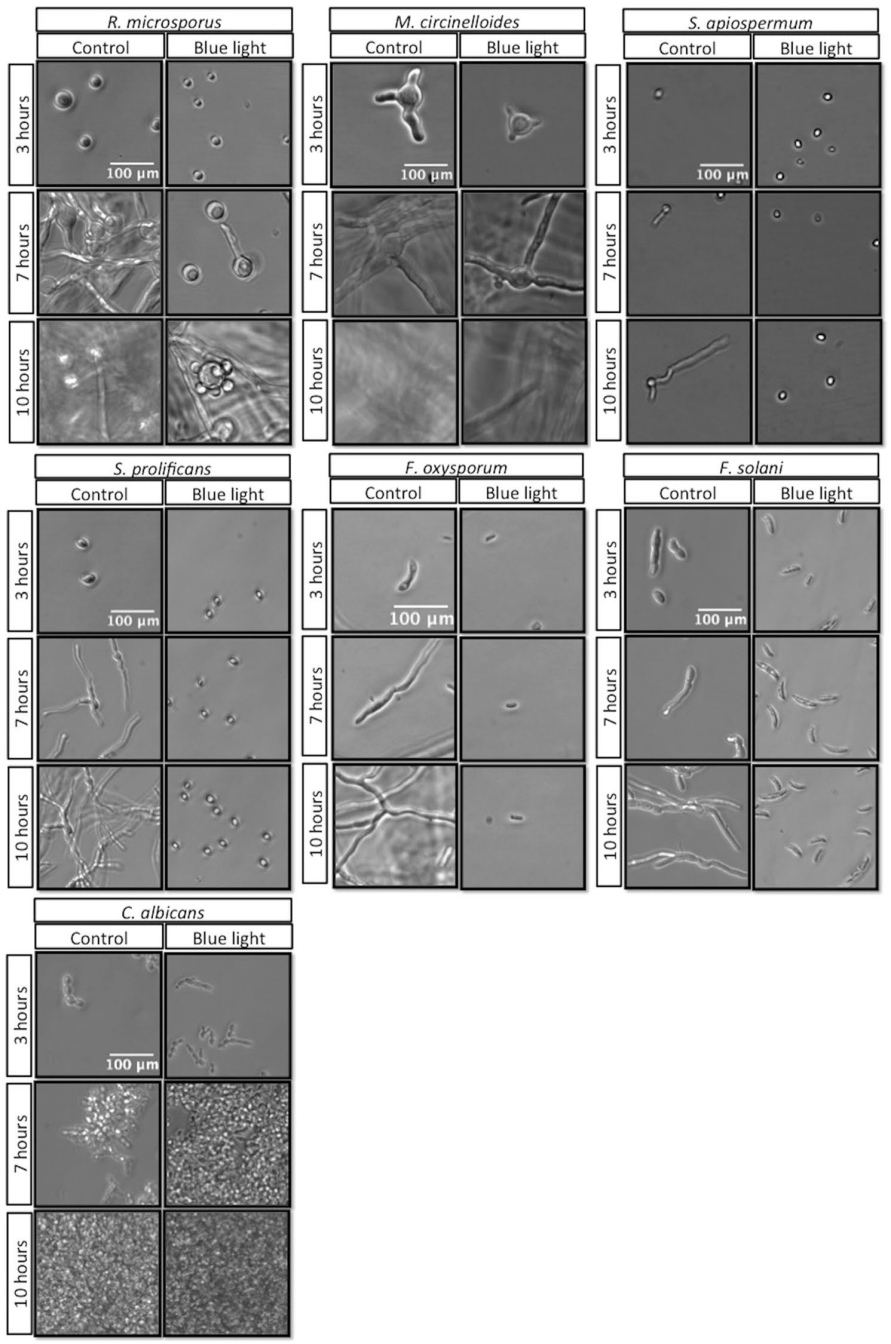
Visualization of blue light effect on fungal spores and cells. Time-lapse imaging shows that blue light 60 min treatment (216J/cm^2^) under controlled temperature conditions is effective against *S*. *prolificans* and *F*. *solani* species but not *C*. *albicans* or Mucorales.

To visualize blue light effects on fungal pathogens we performed time-lapse imaging on treated fungal spores and additionally included *Candida albicans*, which has previously been shown to be sensitive to blue light killing^6^ (Fig. 2). Time lapse imaging demonstrated that blue light treatment permanently inhibited germination of *Scedosporium* and *Fusarium* species, but that *Rhizopus microsporus*, *Mucor circinelloides* and *Candida albicans* eventually recovered full growth capability. Thus blue light induces a germination/growth arrest that appears permanent in most fungi, but only transient in the Mucorales and *Candida* species tested here. It will be of interest in the future to establish whether this pattern is conserved across the diversity of fungal species within these two groups.

Interestingly, during these studies on *Rhizopus microsporus* we also made the chance observation of a morphological change during germination into yeast-like budding (Fig. 2). Further analysis demonstrated that this budding is suppressed by exposure to light (Supplementary Figure 1). Budding of this sort has been previously reported for *M*. *circinelloides* ^8^ but never previously observed in *Rhizopus* species, so this observation raises the possibility that budding in the absence of light stimuli may be widespread within the Mucorales.

### Blue light exposure leads to secondary heating, but this is not a major contributor to the growth inhibition effect

**Figure 3:**
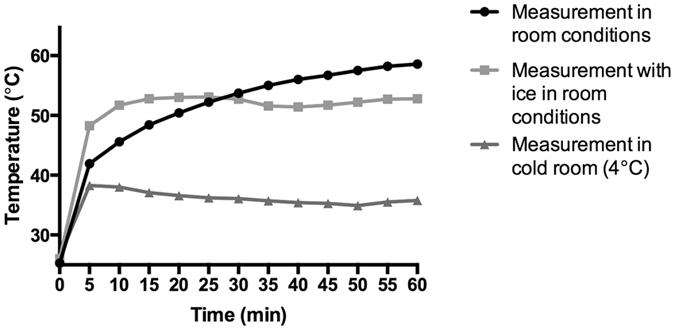
Temperature rises rapidly inside the blue light instrument during operation. Incubating samples on ice within the instrument is insufficient to reduce this effect, but housing the instrument within a cold room (at 4° C) during treatment maintains sample temperatures at 37 °C.

**Figure 4:**
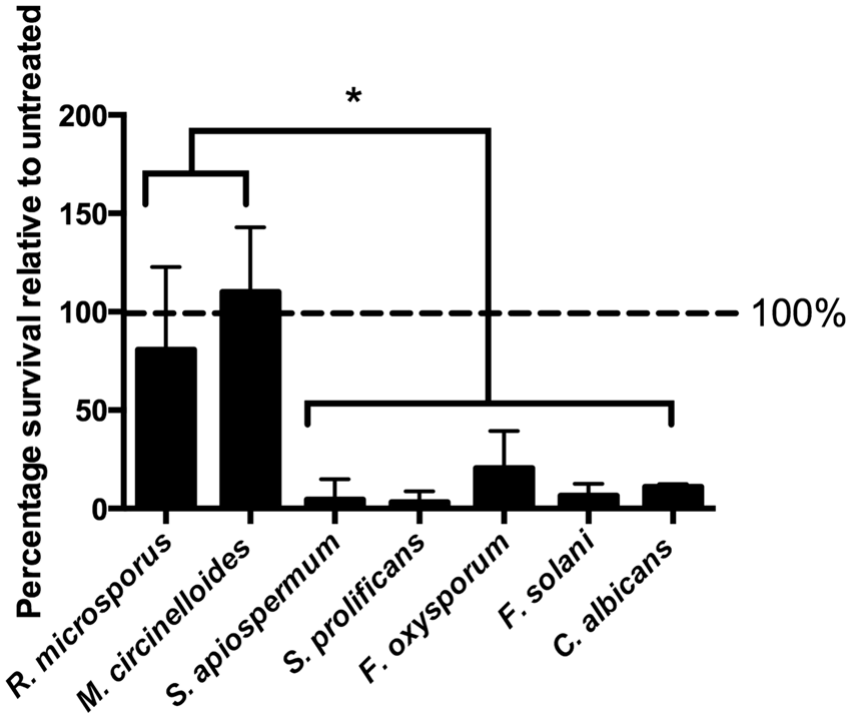
60 min (216J/cm^2^) blue light treatment of fungal spores in cold room conditions shows similar growth inhibition effects. Spores were inoculated in PBS and treated with blue light before plating for growth and CFU enumeration. Error bars represent standard deviation (n = 3, with three experimental replicates). One-way ANOVA followed by Tukey’s multiple comparisons test shows significant difference (p < 0.05 for all comparisons) in blue light treatment survival between *R*. *microsporus* and *M*. *circinelloides* and other species tested.

During our investigations we noted that treated samples were significantly warmer than untreated controls. We therefore measured temperature within the medium for samples within the blue light instrument that were either exposed to blue light or wrapped within foil. In both cases, we noted a very rapid increase in temperature during instrument operation (Fig. 3). Such temperatures are likely to be deleterious to fungal spore survival and we therefore repeated our blue light treatment experiments by housing the instrument within a cold room, which limited the maximum temperature experienced to 37 °C (Fig. 4), a temperature that is fully permissive for growth of these pathogens. When we repeated this assay under these conditions, blue light retained its potent inhibitory effect on the *Fusarium* and *Scedosporium* species and, as before, showed no inhibition of Mucormycete survival (Fig. 4).

### Blue light is highly effective against pre-germinated spores, but also leads to significant cytotoxicity towards mammalian cells

**Figure 5:**
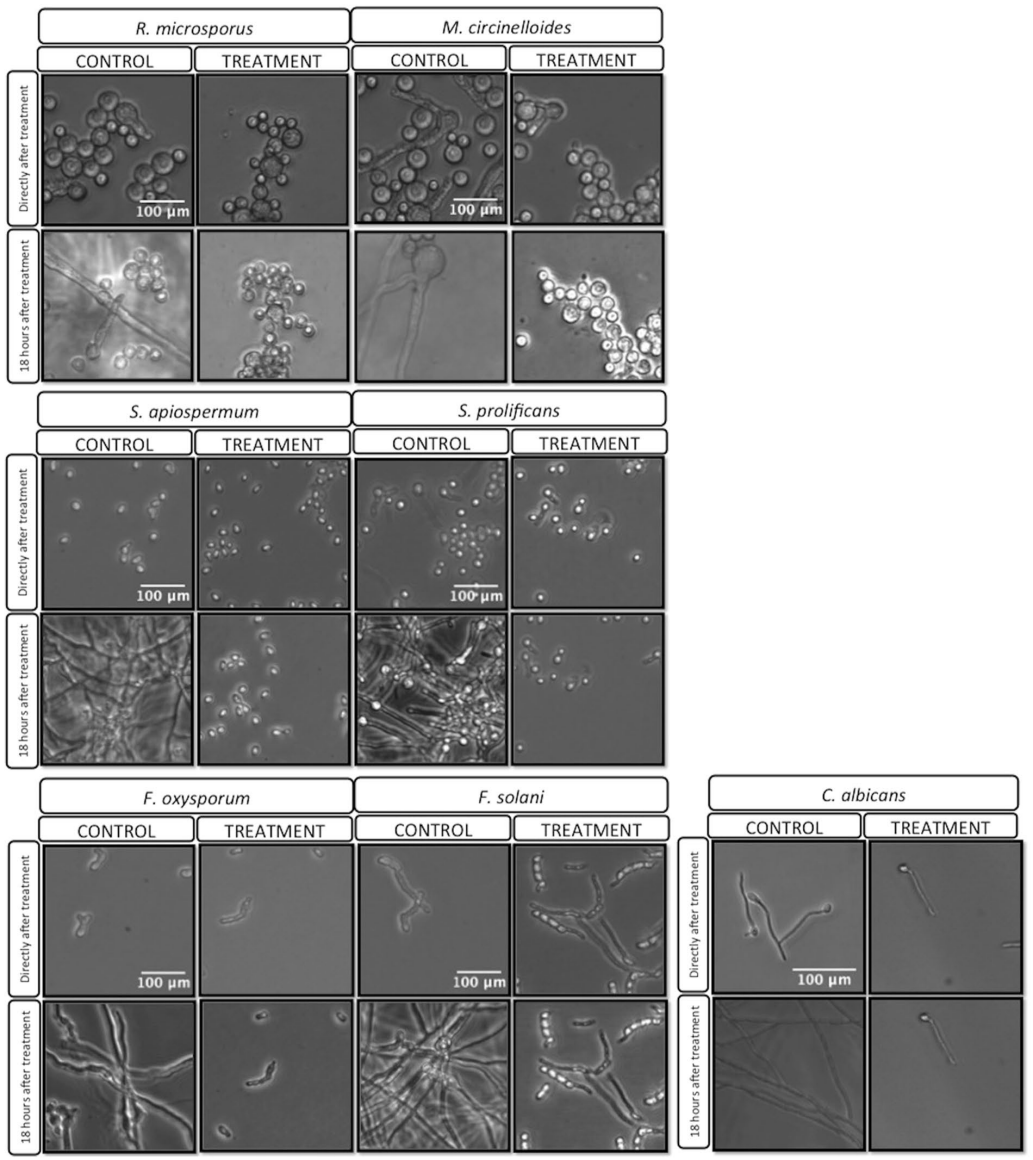
60 min (216J/cm^2^) blue light treatment show antifungal activity on pregerminated spores. Time-lapse imaging shows inhibition of further fungal growth after blue light treatment.

In clinical settings, fungal spores might have germinated (or, in the case of *Candida albicans*, switched from yeast to form hyphae in response to environmental cues such as the presence of serum, low oxygen or high pH) and begun filamentous growth before treatment can be applied. Therefore we investigated the effect of blue light treatment on pre-germinated spores and hyphae. Spores were first germinated for 3–5 hours, and then exposed to 60 minutes of blue light (in cold room conditions to limit temperature exposure to below 37 °C) followed by time-lapse microscopy (Fig. 5). In all cases, blue light treatment effectively stopped further growth of germ tubes, including in Mucormycete species, and *Candida* hyphae, two fungal groups that show resistance to blue light as spores or yeast, respectively (Fig. 1). Thus, blue light is an effective inhibitory treatment for fungal spores that have already germinated, including for species that are resistant to such treatment as spores.

Previous work has demonstrated that blue light can also induce high levels of cell death in mammalian cells^5^. To test whether this was also the case in our system, we J774 murine cells to 15, 30, 45 and 60 min of blue light, under conditions where the temperature was controlled to 37 °C. As previously reported by others for other cell types, blue light exposure led to a rapid, dose-dependent cell death in this cell line whilst J774 cells not exposed to blue light showed undetectable levels of death over the same time period (Supplementary Figure 3).

## Discussion

Fungal infections are a common complication of traumatic injuries sustained in both military and civilian environments like agricultural, motor vehicle, and natural disasters or blunt crush injuries^1^.

Here we have tested the most significant trauma-associated fungi, including Mucorales, *Scedosporium*, *Fusarium* and *Candida* spp. against blue light therapy. Fungal tissue infections are very difficult to treat as many of these species show intrinsic resistance to antifungals and drug accessibility to wounded tissue is poor. Thus blue light may represent a novel approach for dealing with such infections.

We have demonstrated that 60 minutes of blue light treatment, providing an equivalent total dosage of 216J/cm^2^, shows potent inhibition of fungal growth for spores that have already germinated and produced hyphae or germ tubes in all species tested. In addition, such an approach can also be an effective decontaminant of ungerminated spores for most, but not all, pathogenic species.

However, we also note some important caveats to this approach. Firstly, for resistant Mucormycete species, blue light treatment appears to counteract the effect of high-temperature and enhance subsequent germination (Fig. 1) and morphological switching (Fig. 2), the immunological consequences of which remain unknown. This likely reflects the previously characterized role of blue wavelengths as a regulator of fungal growth^9^. In an analogous context, we note that others have previously demonstrated the ability of blue light to enhance virulence in selected bacterial pathogens, such as Brucella species^10^. Thus blue light may inadvertently work against other forms of fungal decontamination (such as heat treatment) in resistant species. Secondly, we note that the high-intensity blue light system used here generates considerable heat - a factor that is important to control for when assessing its efficacy against pathogenic microbes. The extent to which secondary heating occurs with other blue light instruments is unknown, but should be borne in mind as a potential confounding factor in other studies. Lastly, although (unlike ultraviolet) blue light is not mutagenic^11^, both our own studies (Supplementary Figure 3) and previous work^5^ demonstrate relatively high levels of toxicity for blue light against some mammalian cells - an observation that may limit the application of this approach directly to patients. Thus blue light offers a potentially useful antimicrobial approach, but its toxicity towards mammalian cells and its limited efficacy against some fungal spores may suggest it is more appropriately used as a selective surface decontaminant than an *in vivo* antifungal.

## Materials and Methods

### Fungal strains

Eight different strains were tested: *Rhizopus microsporus* 12.6652333 and *Mucor circinelloides* NRRL3631^12^, *Scedosporium apiospermum* IHEM 14462, *Scedosporium prolificans* IHEM 5608, *Fusarium oxysporum* IHEM 25499, *Fusarium solani* IHEM 6092, all from the Belgian Coordinated Collections of Microorganisms, as well as *Candida albicans* SN152^13^. Mucorale strains were grown on Sabouraud agar plates, *Scedosporium* and *Fusarium* spp. were grown on Potato Glucose agar plates for at least 10 days before use at room temperature, and *Candida* was grown on Yeast Extract Peptone agar plates for 1 day at 37° C.

### Media

Spores were washed off agar plates with phosphate-buffered saline (PBS) (Fisher Scientific), then spun down and resuspended in PBS for cell counting in a haemocytometer. For *Candida* species, a single yeast colony was picked from YPD agar using an inoculation loop and inoculated in YPD or RPMI broth (depending on the experiment) for counting in a haemocytometer. For blue light testing, fungal spores and cells were inoculated into PBS and exposed to blue light, then transferred to appropriate agar plates after treatment for subsequent colony counting. For microscopy experiments, fungal spores and cells were inoculated in Sabouraud broth (Sigma Aldrich).

### Temperature measurement

The temperature of treated cell cultures was measured within 24 well plates using a submersible aquarium thermometer (ETI, UK). The temperature was recorded constantly throughout the treatment and plotted at five minutes intervals. This temperature measurement was repeated on three separate occasions over of period of three months, with extremely consistent data on each occasion.

### Treatment with blue light

A LED flood array^14^, composed of 144 LEDs (Henkel-Locite, Hemel Hampstead, UK), was used to treat a 10 × 10 cm area using high-intensity blue light (405 nm). All experiments on fungal cells were performed by placing samples within the treatment area for 1 hour, providing an equivalent total dosage of 216J/cm^2^. To control for temperature effects, experiments were either performed in a cold room (4 °C) or at room temperature, as described. Fungal cells were inoculated into PBS and then placed under the treatment area for 60 minutes (216J/cm^2^). Two control conditions were used: one plate covered with aluminum foil inside the blue light machine (exposed to temperature, but not blue light, effects) and one kept outside the instrument during operation (not exposed to either raised temperatures or blue light). Following treatment, each fungal suspension was transferred onto appropriate agar plates and CFU numbers were counted following growth (typically a few days later). Each experiment was performed in technical triplicate and repeated on at least three occasions. For determining an effect of blue light on fungal morphology assay, fungal spores were inoculated onto agar and treated with blue light for 1 hour in the room temperature then left to grow for a few days, or agar plates were treated with blue light for 1 hour, then fungal spores/cells were inoculated on it for growth. For determining blue light cytotoxicity, the murine cell line J774 was treated with blue light for 15, 30, 45 and 60 min in cold room to control for excessive heating. Following treatment cells were stained with Trypan blue for viability and counted using a haemocytometer to determine percentage survival (Supplementary Figure 3). J774 cell line was incubated in complete DMEM (Sigma-Aldrich), phenol free, plus 100U/ml of penicillin and 100 U/ml of streptomycin (Sigma), 2 mM l- glutamine (Sigma), and 10% fetal bovine serum (FBS) (Sigma) in 37°C and CO2 condition.

### Time-lapse imaging

Time-lapse imaging was performed on fungal spores immediately after treatment with blue light, using a Nikon Eclipse Ti microscope with a long working distance (LWD) 0.53 20 × objective for 18 h with 5 min intervals. Movies for publication were analysed and prepared in NIS Element software.

### Light controls for *R*. *microsporus*

Light controls for *R*. *microsporus* and *M*. *circinelloides* NRRL3631 were performed on fungal spores that were washed off the agar plates, inoculated in Sabouraud broth (Sigma Aldrich) and covered with aluminum foil or exposed to light. Plates were incubated at room temperature for 10 hours and samples were taken for pictures.

## Electronic supplementary material

Supplementary Figures
