# Supplementary material for: Species-specific antifungal activity of blue light
Source: Sci Rep. 2017 Jul 4;7:4605. doi: 10.1038/s41598-017-05000-0 (PMC5496878; doi:10.1038/s41598-017-05000-0)

Species-specific antifungal activity of blue light

Wioleta J. Trzaska[ab], Helen E. Wrigley[a], Joanne E. Thwaite[c], Robin C. May[ab*]

Institute of Microbiology and Infection and School of Biosciences, University of Birmingham, United Kingdom[a], NIHR Surgical Reconstruction and Microbiology Research Centre, University Hospitals of Birmingham NHS Foundation Trust, Queen Elizabeth Hospital, Birmingham, United Kingdom[b]
Chemical, Biological and Radiological Division, DSTL, Porton Down, Salisbury, Wiltshire, UK[c]
Correspondence to r.c.may@bham.ac.uk

Supplementary Figure 1. Mucorales switch their morphology to budding-like yeast in the absence of light exposure. Both *R. microsporus and M. circinelloides* NRRL3631 were washed off the plates and inoculated in Sabouraud broth for 10 hours. One sample was exposed to light while the second covered with aluminum foil. Both strains switch their morphology from filamentation to a yeast-like budding when deprived of light during germination-conducive conditions.

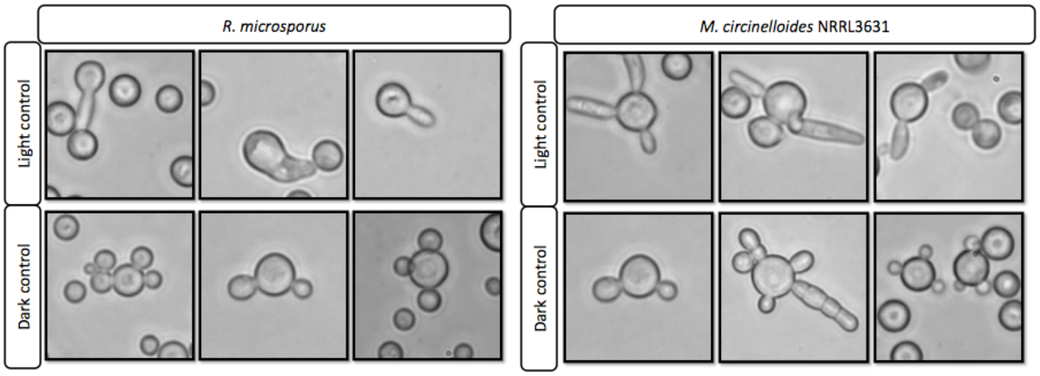

Supplementary Figure 2. Agar plates were treated with blue light (BL) for 60min prior inoculation of fungal spores/cells. There are no differences in fungal growth between agar pre-treated with blue light and control plates.

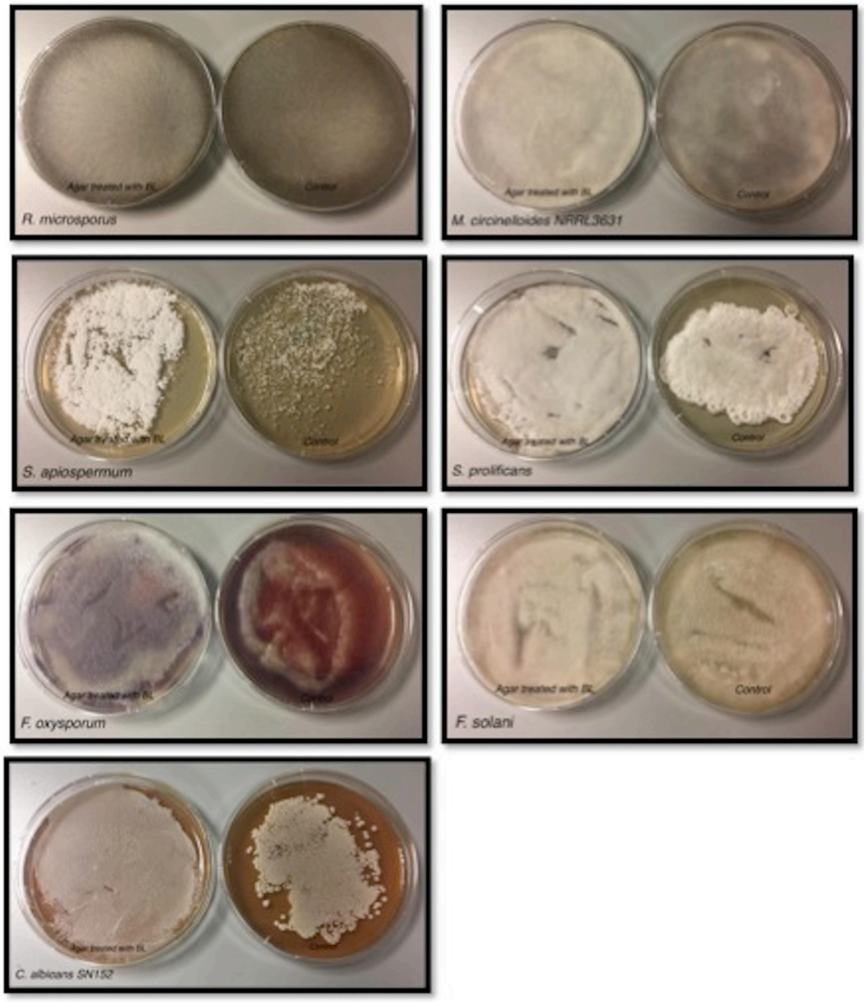

Supplementary Figure 3. 60min blue light treatment of J774 murine cell line in cold room. Error bars represent standard deviation (n=3, with three experimental replicates).

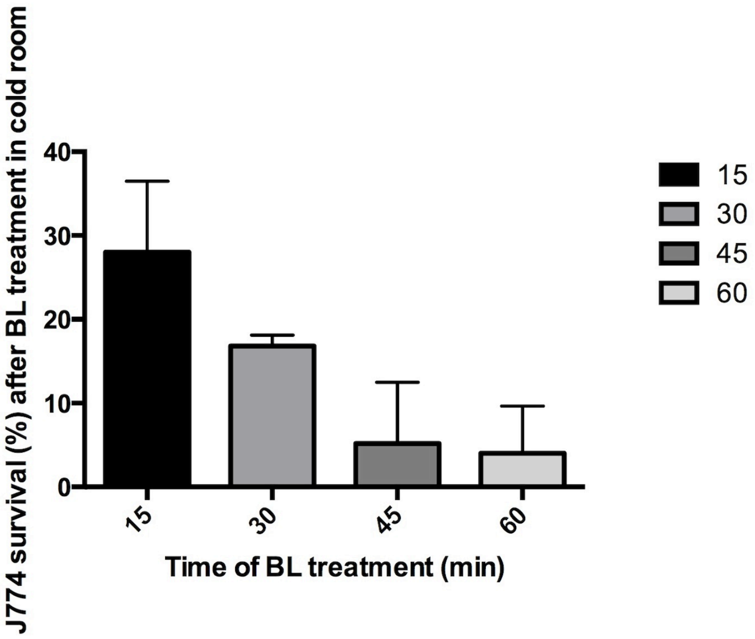

Supplement: Supplementary file 1 — Supplementary Figures [file 41598_2017_5000_MOESM1_ESM.pdf]
